# Supplementary material for: Antibiotic resistance selection and deselection in municipal wastewater from 47 countries
Source: Nat Commun. 2025 Nov 3;16:9698. doi: 10.1038/s41467-025-65670-7 (PMC12583516; doi:10.1038/s41467-025-65670-7)
Supplement: Supplementary file 1 — Supplementary Information [file 41467_2025_65670_MOESM1_ESM.pdf]

Supplementary Information for

Antibiotic resistance selection and deselection in municipal wastewater from 47 countries

Zhuofeng Yu [1,2], Declan A. Gray [1,2,3], Jerker Fick [4], Noel Waters [1,2], Richard Lindberg [4], Roman Grabic [5], Mats Tysklind [4], Mutshiene Deogratias Ekwanzala [1,2], Hannah-Marie Martiny [6], Carl-Fredrik Flach [1,2], Frank M. Aarestrup [6], D. G. Joakim Larsson [1,2,#]

[1] Centre for Antibiotic Resistance Research (CARe) at the University of Gothenburg, Gothenburg, Sweden

[2] Department of Infectious Diseases, Institute of Biomedicine, The Sahlgrenska Academy at the University of Gothenburg, Gothenburg, Sweden

[3] The Sahlgrenska University Hospital, Gothenburg, Sweden

[4] Department of Chemistry, Umea University, Umea, Sweden

[5] South Bohemian Research Center of Aquaculture and Biodiversity of Hydrocenoses, Faculty of Fisheries and Protection of Waters, University of South Bohemia in České Budějovice, Vodňany, Czech Republic

[6] Research Group for Genomic Epidemiology, Technical University of Denmark, Kgs. Lyngby, Denmark

[#] Corresponding Author.

E-mail: joakim.larsson@fysiologi.gu.se.

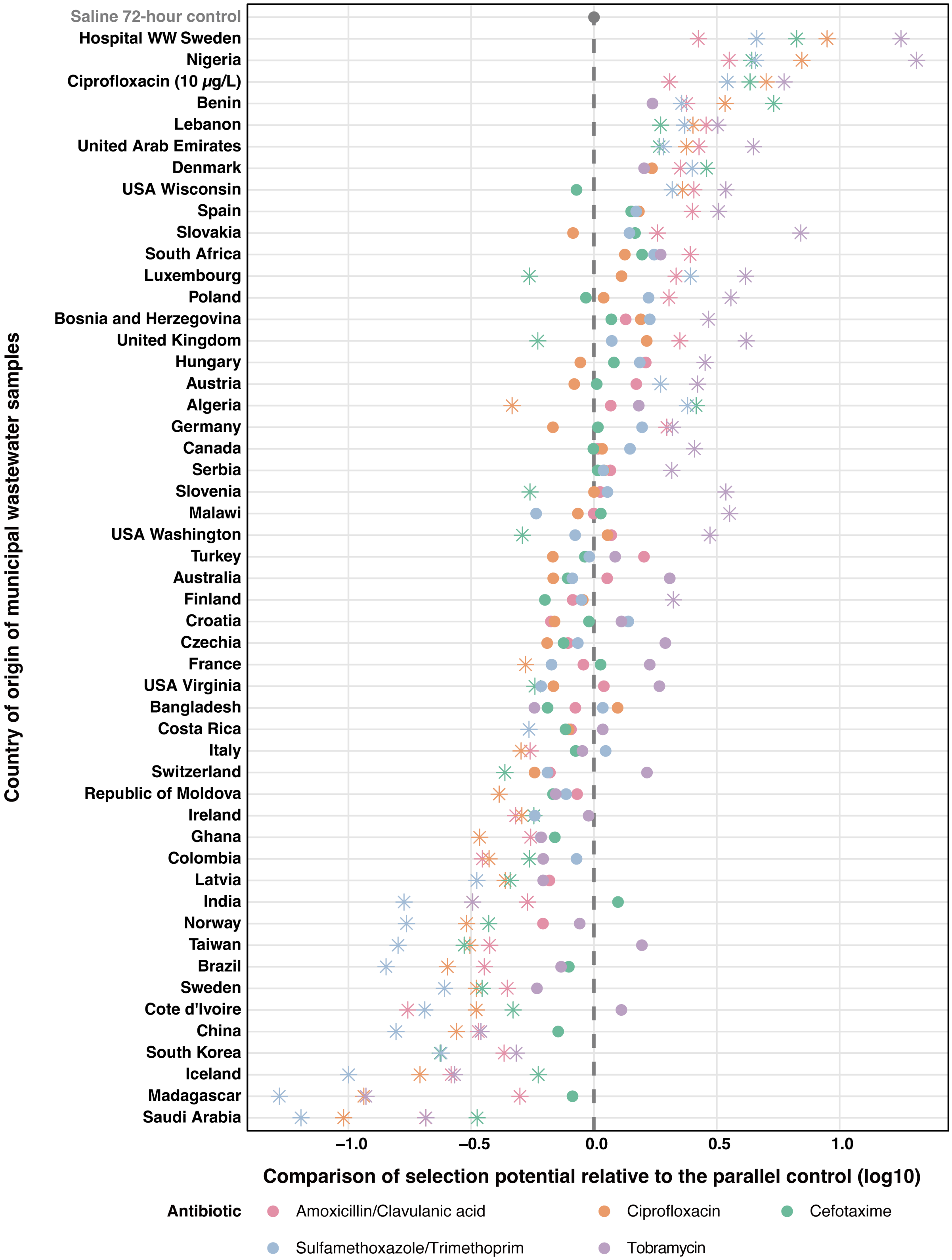

Supplementary Fig. 1 | Selection potential of globally sourced municipal wastewater for different antibiotic resistances compared to the parallel 72-hour saline control.

The selection potential represents the %resistance in an *Escherichia coli* community exposed to globally sourced wastewater samples [and ciprofloxacin (10 μg/L) and wastewater from a Swedish hospital as positive controls] after three passages (72-hour) relative to the %resistance of saline after three passages (72-hour, parallel control; dashed grey line). The selection potential of individual resistances relative to the parallel control is log10-transformed to allow resistance to different antibiotics to be viewed at a comparable scale. Data points to the right of the control indicate "increased persistence" [1], whereas those to the left indicate decreased persistence [1]. Countries and positive controls are ordered based on the mean selection potential across all antibiotics. Three samples were collected from locations in the United States of America (states indicated). Selection potentials that are not significantly different from baseline are depicted as solid circles, while those with statistically significant differences ($p < 0.05$, two-sided Wald test with Benjamini-Hochberg adjustment; see Supplementary Data 2 for the detailed p values) are marked with asterisks.

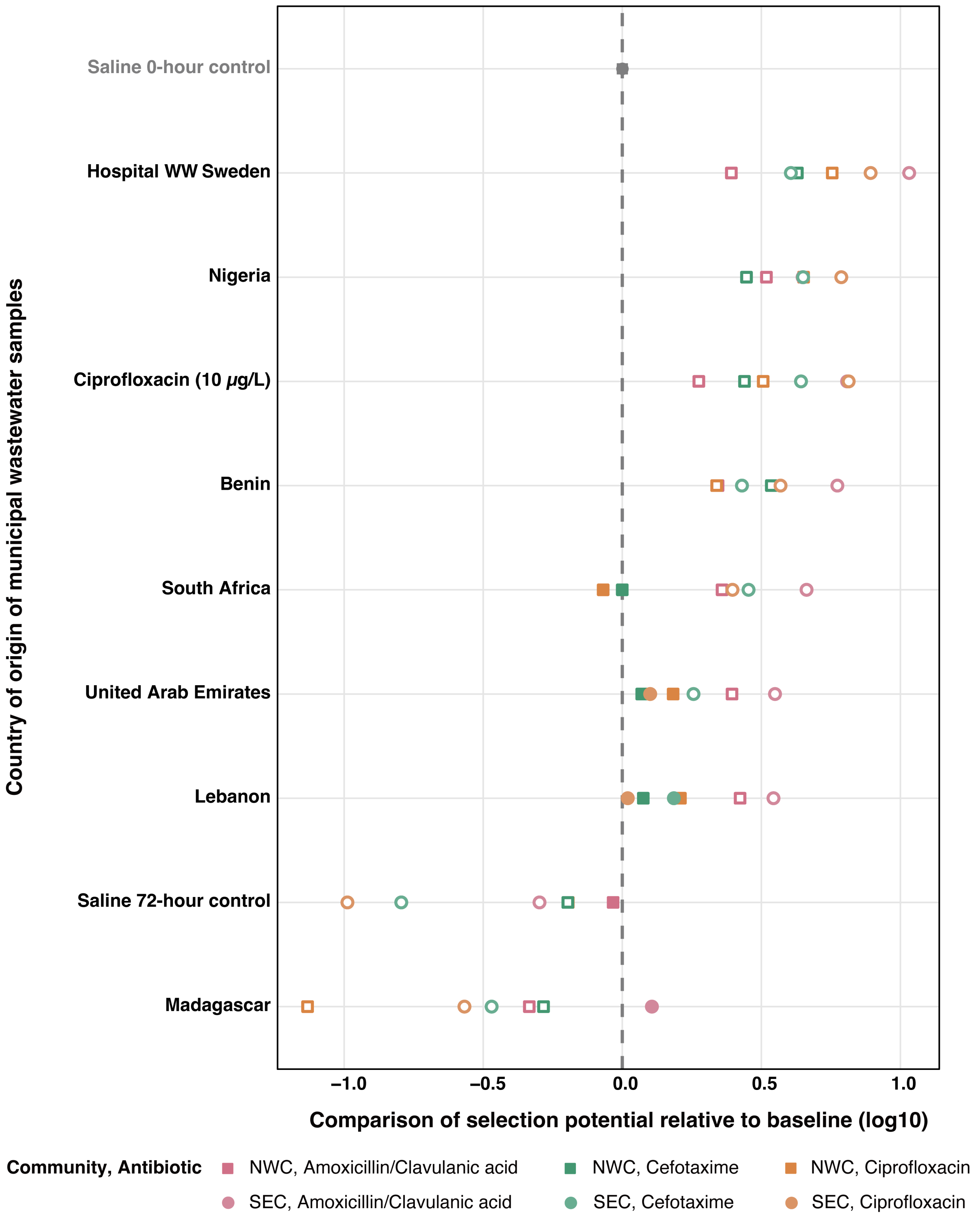

Supplementary Fig. 2 | Comparison of selection potential of globally sourced municipal wastewater in *E. coli* assessed using either single- or multi-species communities.

The selection potential represents the %resistance in an *E. coli* community exposed to globally sourced wastewater samples [and ciprofloxacin (10 μg/L) and wastewater from a Swedish hospital as positive controls, and saline as negative control] after three passages (72-hour) relative to the initial %resistance (0-hour, baseline; dashed grey line). Selection data were derived from experiments exposing either a synthetic *E. coli* community or a natural wastewater community to sterile-filtered municipal wastewater and control samples. "NWC" represents the data for the natural wastewater community and is marked as squares; "SEC" represents the data for the synthetic *E. coli* community and is marked as circles. The selection potential of individual resistances relative to baseline is log10-transformed to allow resistance to different antibiotics to be viewed at a comparable scale. Data points to the right of the baseline indicate positive selection, whereas those to the left indicate deselection. Countries and controls are ordered based on the mean selection potential across all antibiotics. Selection potentials that are not significantly different from baseline (0-hour) are depicted as solid circles, while those with statistically significant differences ($p < 0.05$, two-sided Wald test with Benjamini-Hochberg adjustment; see Supplementary Data 1 and Supplementary Data 3 for the detailed *p* values) are marked with asterisks.

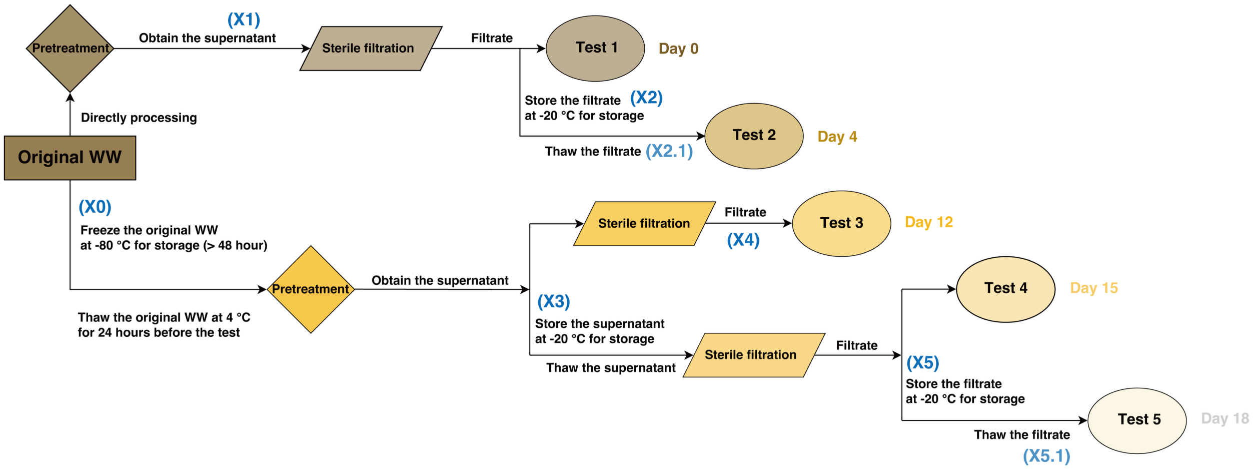

Supplementary Fig. 3 | Flowchart for the assessment of sample processing and storage effects on selection potential.

X0 to X5 represent samples taken at different time points during the processing of a mock sample to assess how processing and storage conditions affect selection potential (Tests 1 – 5) and antibiotic concentrations. "Original WW" refers to the mock sample, which is a fresh 24-hour composite hospital wastewater sample collected from the Sahlgrenska University Hospital, Gothenburg, Sweden, on October 26, 2023. During the pretreatment step, a centrifugation (10,000 *g*, 10 min, 4 °C) was performed. Sterile filtration was conducted using 0.22 μm pore size S-Pak filters (Millipore, Bedford, USA).

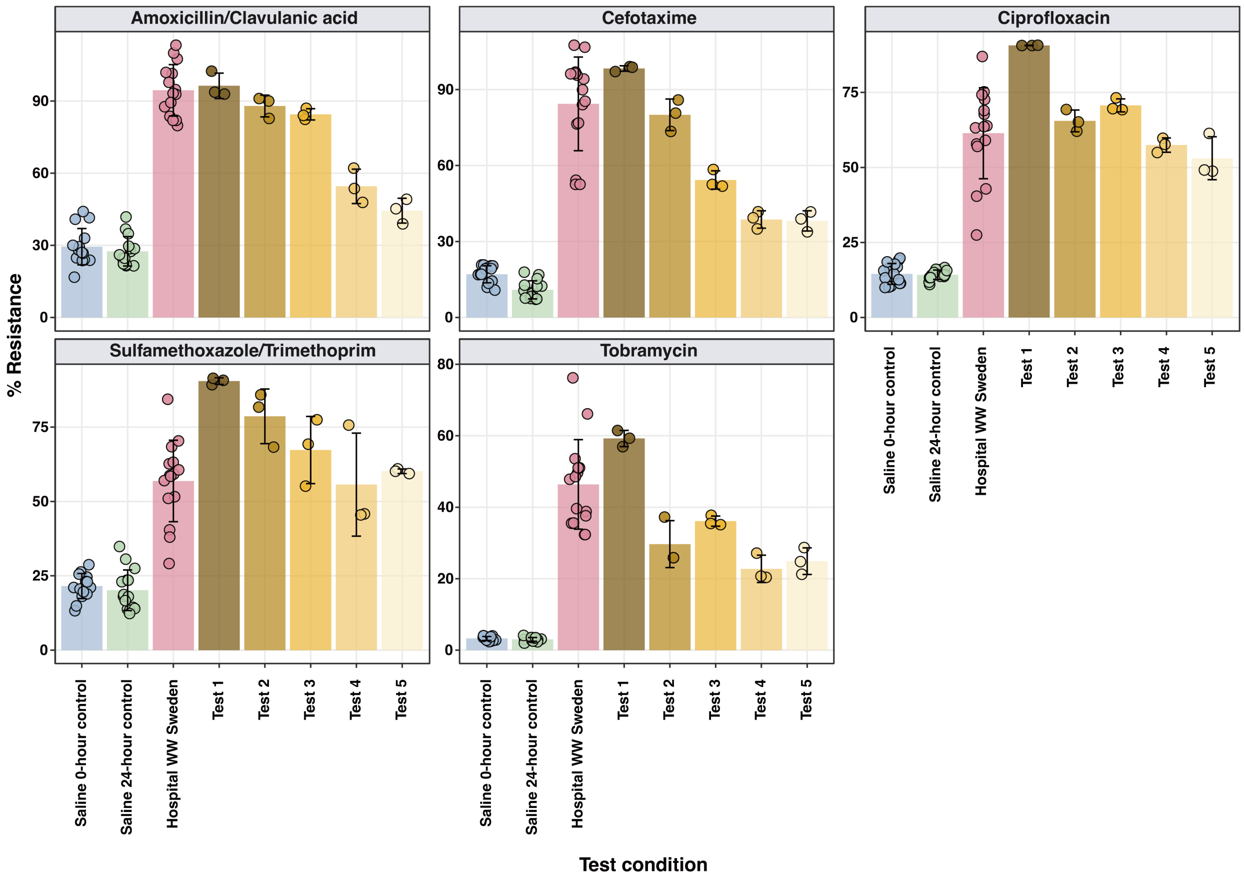

Supplementary Fig. 4 | Variation in selection potential throughout sample processing.

Bars represent the %resistant *E. coli* to antibiotics in a synthetic *E. coli* community after 24-hour exposure in the presence of hospital wastewater samples at different processing time points (Test 1 – Test 5), along with control conditions [saline (negative control) and wastewater from a Swedish hospital ("Hospital WW Sweden"; collected on April 28, 2022; positive control)], see also Supplementary Fig. 3. Data are presented as mean values ± standard deviations. Specifically, the error bars indicate the standard deviation across three replicates for Test 1 – Test 5, and 15 replicates for the saline and Hospital WW Sweden as controls are performed in every test. Data points are shown in each test condition and control, representing the direct measurements from the replicates.

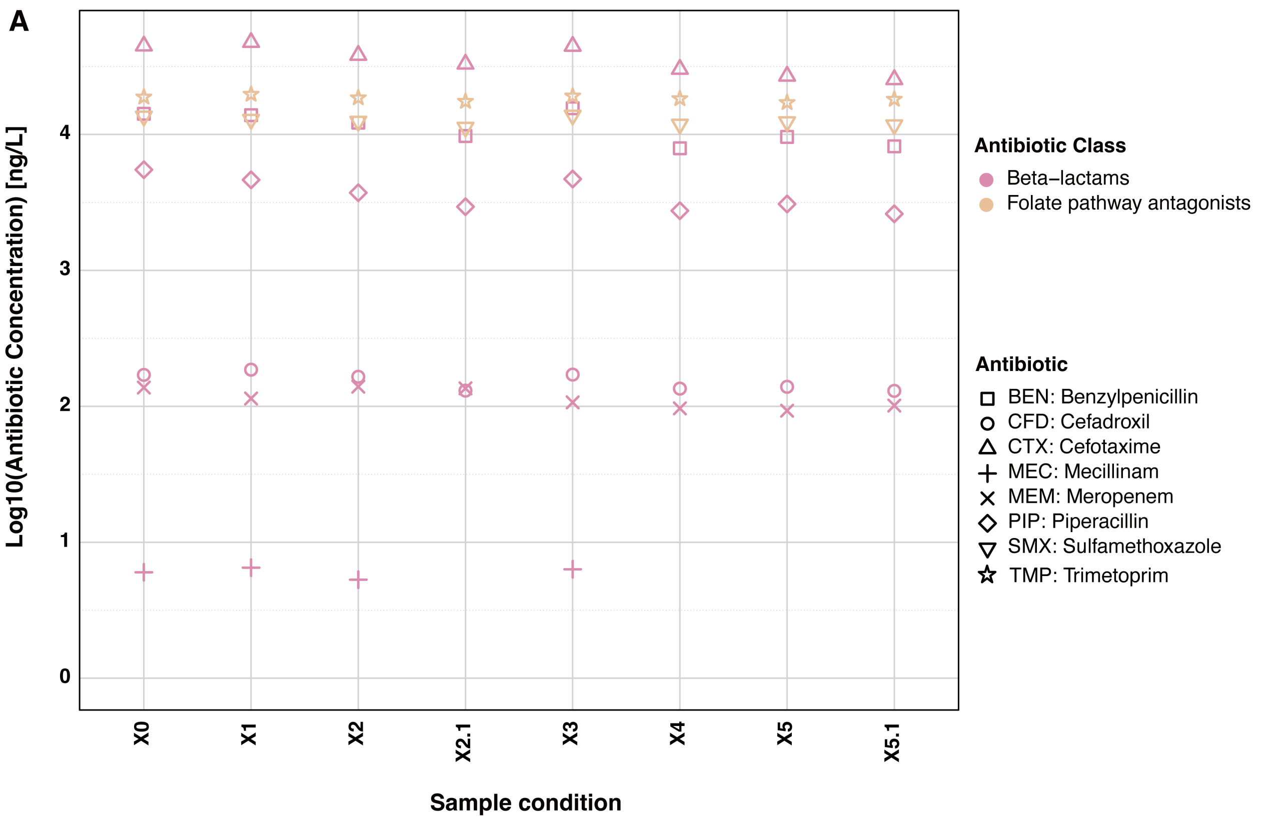

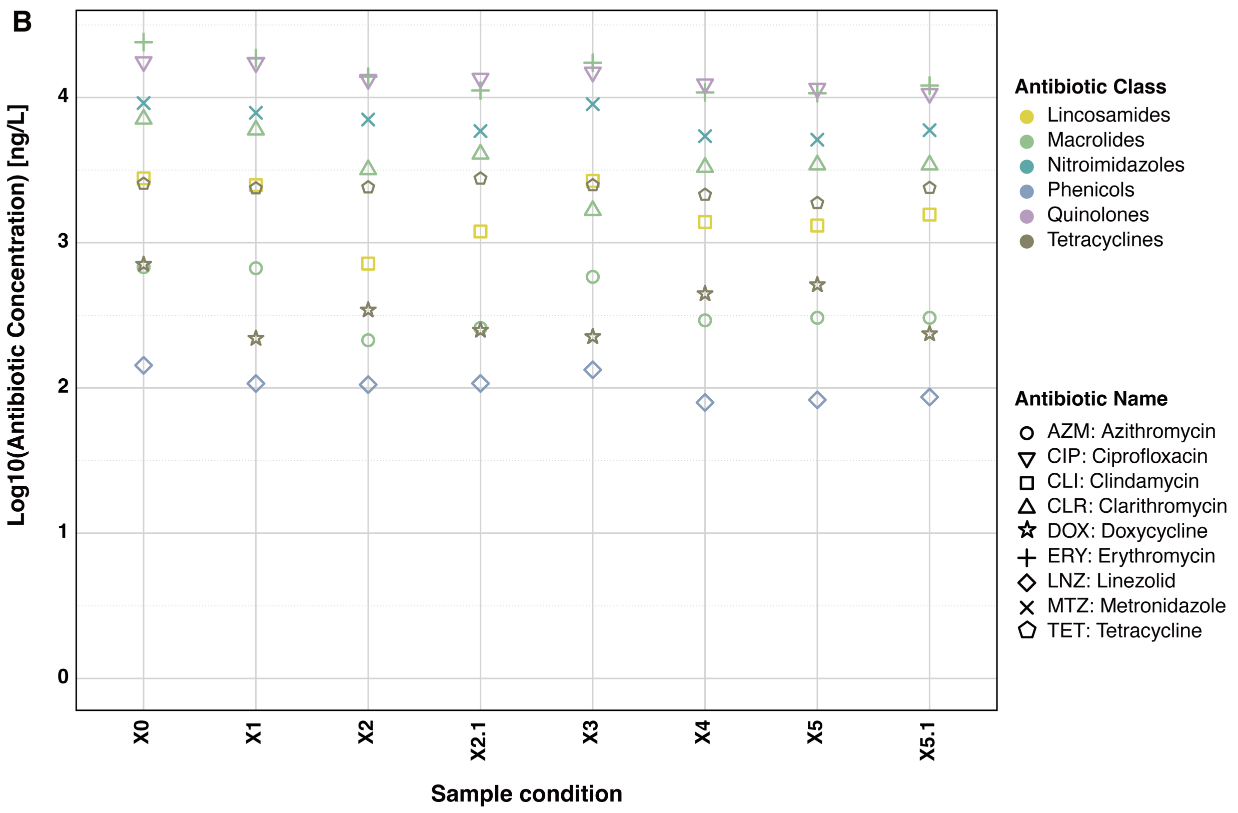

Supplementary Fig. 5 | Variation in antibiotic concentrations throughout sample processing.

A Antibiotic concentrations (ng/L) of beta-lactams (ampicillin, benzylpenicillin, cefadroxil, cefotaxime, clavulanic acid (inhibitor), mecillinam, meropenem, piperacillin, pivmecillinam) and folate pathway antagonists (sulfamethoxazole, trimetoprim). **B** Antibiotic concentrations of lincosamides (clindamycin), macrolides (azithromycin, clarithromycin, erythromycin), nitroimidazoles (metronidazole), phenicols (linezolid), quinolones (ciprofloxacin, norfloxacin) and tetracyclines (doxycycline, oxytetracycline, tetracycline). Different antibiotics are represented by distinct symbols. The colour scheme denotes distinct antibiotic classes. Details for the sampling conditions (X0 to X5) are explained in Supplementary Fig. 3.

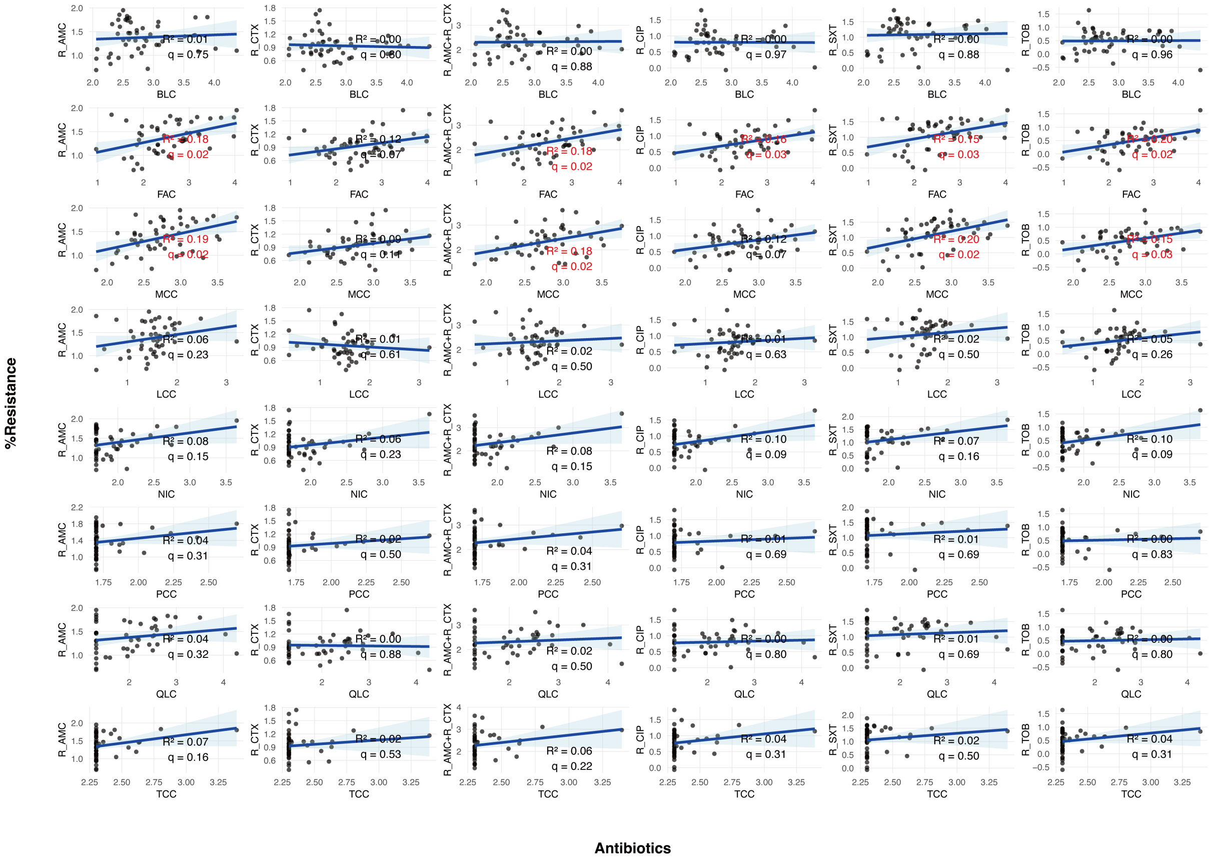

Supplementary Fig. 6 | Regression analysis of antibiotic concentrations (by class) against selection potential in globally sourced municipal wastewater samples.

Statistical significance is determined using Benjamini-Hochberg adjusted *p* values (denoted as *q* values) at a threshold of < 0.05. Significant associations ($q < 0.05$) are highlighted in red text in the scatterplots. Data presented here have been log10-transformed.

Abbreviations

1. Antibiotic concentrations: "BLC", beta-lactam class antibiotic concentration; "FAC", folate pathway antagonist class antibiotic concentration; "MCC", macrolide class antibiotic concentration; "LCC", lincosamide class antibiotic concentration; "NIC", nitroimidazole class antibiotic concentration; "PCC", phenicol class antibiotic concentration; "QLC", quinolone class antibiotic concentration; "TCC", tetracycline class antibiotic concentration.

2. Selection potential (%resistance): “R_AMC”, observed %resistance after exposure for amoxicillin/clavulanic acid; “R_CTX”, observed %resistance after exposure for cefotaxime; “R_AMC+R_CTX”, sum of observed %resistance after exposure for amoxicillin/clavulanic acid and cefotaxime; “R_CIP”, observed %resistance after exposure for ciprofloxacin; “R_SXT”: observed %resistance after exposure for sulfamethoxazole/trimethoprim; “R_TOB”, observed %resistance after exposure for tobramycin.

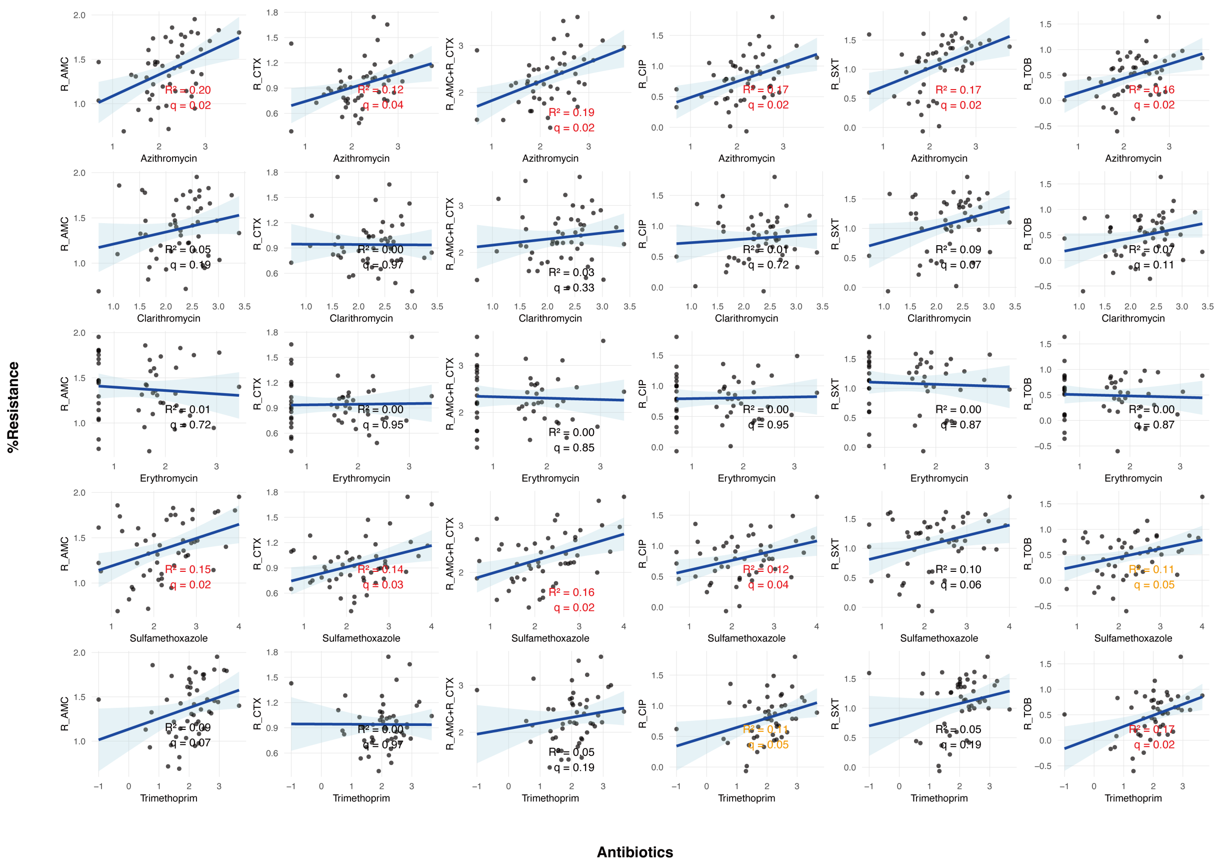

Supplementary Fig. 7 | Regression analysis of folate pathway antagonist and macrolide concentrations against selection potential in globally sourced municipal wastewater samples.

Statistical significance is determined using Benjamini-Hochberg adjusted *p* values (denoted as *q* values) at a threshold of < 0.05. Significant associations ($q < 0.05$) are highlighted in red text in the scatterplots. Associations at the threshold ($q = 0.05$) are highlighted in orange text, representing findings at the borderline of statistical significance. Data presented here have been log10-transformed.

Abbreviations

Selection potential (%resistance): "R_AMC", observed %resistance after exposure for amoxicillin/clavulanic acid; "R_CTX", observed %resistance after exposure for cefotaxime; "R_AMC+R_CTX", sum of observed %resistance after exposure for amoxicillin/clavulanic acid and cefotaxime; "R_CIP", observed %resistance after exposure for ciprofloxacin; "R_SXT": observed %resistance after exposure for

sulfamethoxazole/trimethoprim; “R_TOB”, observed %resistance after exposure for tobramycin.

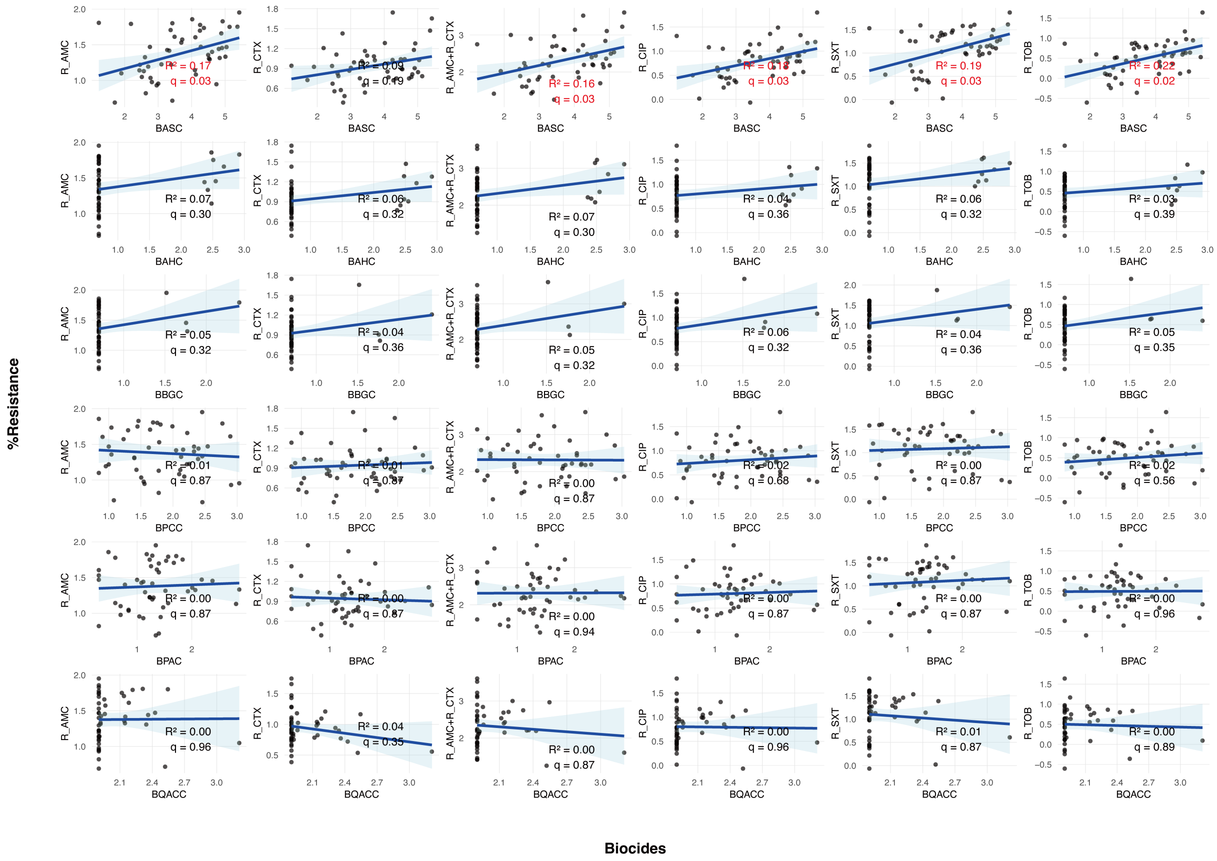

Supplementary Fig. 8 | Regression analysis of biocide concentrations (by class) against selection potential in globally sourced municipal wastewater samples.

Statistical significance is determined using Benjamini-Hochberg controlled *p* values (denoted as *q* values) at a threshold of < 0.05. Significant associations ($q < 0.05$) are highlighted in red text in the scatterplots. Data presented here have been log10-transformed.

Abbreviations

1. Biocide concentrations: “BASC”, biocide acid class concentration; “BAHC”, biocide aldehyde class concentration; “BBGC”, biocide biguanide class concentration; “BPCC”, biocide phenolic compound class concentration; “BPAC”, biocide polyamine class concentration; “BQACC”, biocide quaternary ammonium compound class concentration.

2. Selection potential (%resistance): “R_AMC”, observed %resistance after exposure for amoxicillin/clavulanic acid; “R_CTX”, observed %resistance after exposure for cefotaxime; “R_AMC+R_CTX”, sum of observed %resistance after exposure for amoxicillin/clavulanic acid and cefotaxime; “R_CIP”, observed %resistance after exposure for ciprofloxacin; “R_SXT”: observed %resistance after exposure for sulfamethoxazole/trimethoprim; “R_TOB”, observed %resistance after exposure for tobramycin.

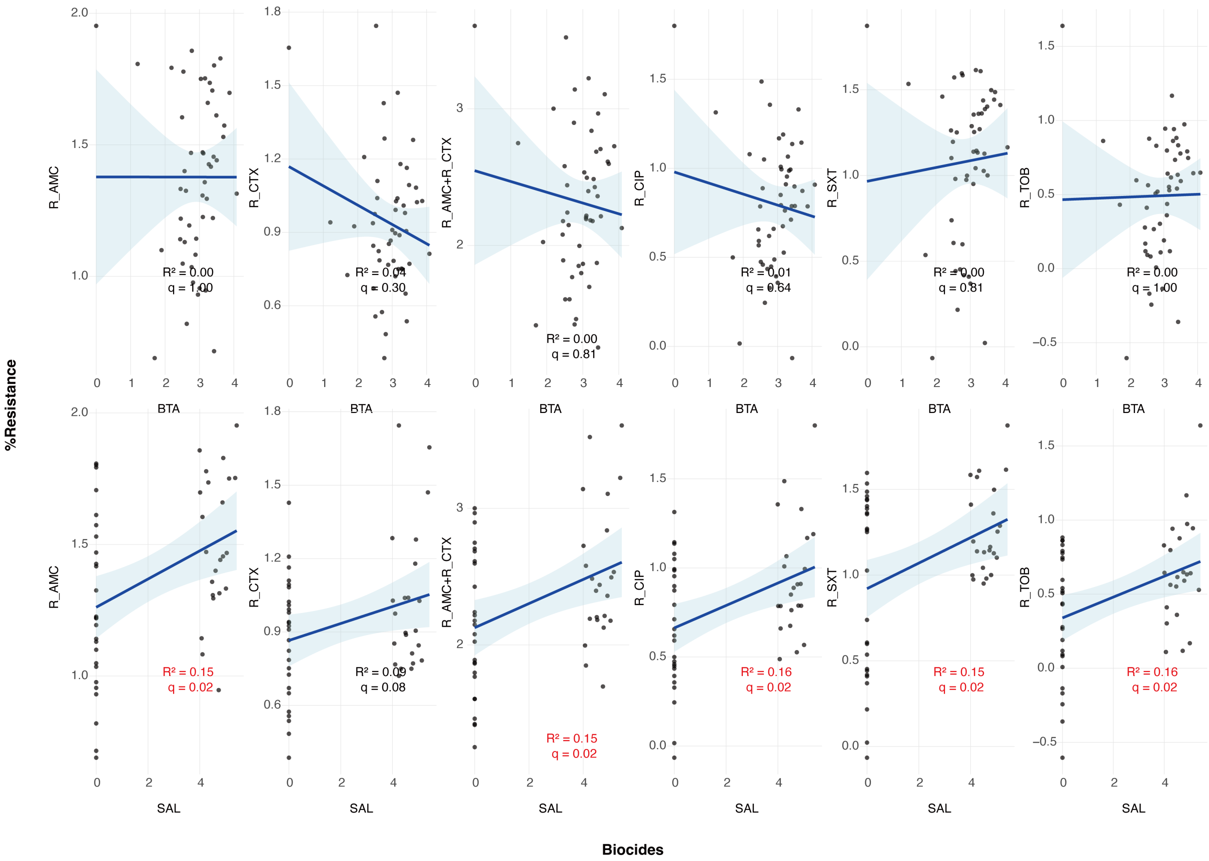

Supplementary Fig. 9 | Regression analysis of biocide acid concentrations against selection potential in globally sourced municipal wastewater samples.

Statistical significance is determined using Benjamini-Hochberg controlled *p* values (denoted as *q* values) at a threshold of < 0.05. Significant associations ($q < 0.05$) are highlighted in red text in the scatterplots. Data presented here have been log10-transformed.

Abbreviations

1. Biocide acid concentrations: "BTA", 1H-benzotriazole; "SAL", salicylic acid.

2. Selection potential (%resistance): "R_AMC", observed %resistance after exposure for amoxicillin/clavulanic acid; "R_CTX", observed %resistance after exposure for cefotaxime; "R_AMC+R_CTX", sum of observed %resistance after exposure for amoxicillin/clavulanic acid and cefotaxime; "R_CIP", observed %resistance after exposure for ciprofloxacin; "R_SXT": observed %resistance after exposure for

sulfamethoxazole/trimethoprim; “R_TOB”, observed %resistance after exposure for tobramycin.

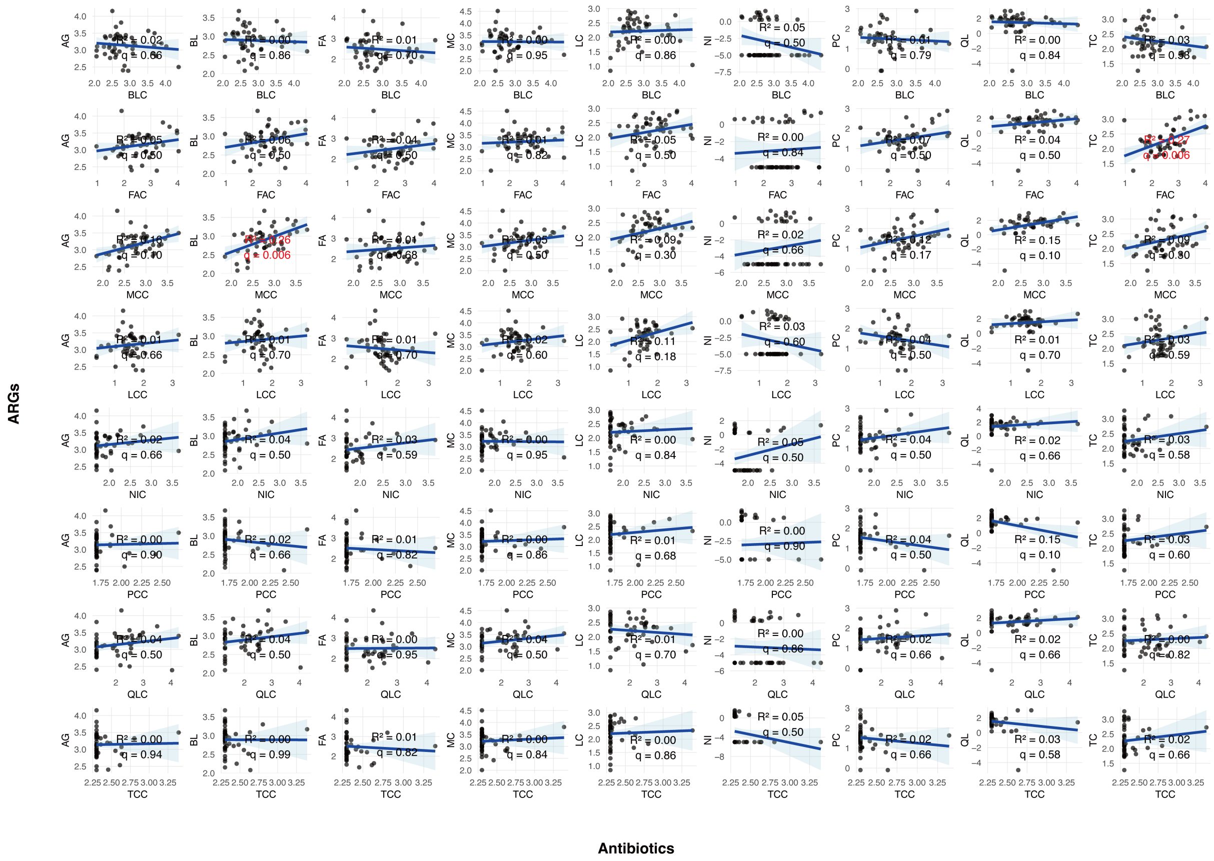

Supplementary Fig. 10 | Regression analysis of antibiotic concentrations (by class) against ARG relative abundances (by class) in globally sourced municipal wastewater samples.

Statistical significance is determined using Benjamini-Hochberg controlled *p* values (denoted as *q* values) at a threshold of < 0.05. Significant associations ($q < 0.05$) are highlighted in red text in the scatterplots. Data presented here have been log10-transformed.

Abbreviations

1. Antibiotic concentrations: "BLC", beta-lactam class antibiotic concentration; "FAC", folate pathway antagonist class antibiotic concentration; "MCC", macrolide class antibiotic concentration; "LCC", lincosamide class antibiotic concentration; "NIC", nitroimidazole class antibiotic concentration; "PCC", phenicol class antibiotic concentration; "QLC", quinolone class antibiotic concentration; "TCC", tetracycline class antibiotic concentration.

2. ARG relative abundances: “AG”: aminoglycoside class resistance gene relative abundance; “BL”, beta-lactam class resistance gene relative abundance; “FA”, folate pathway antagonist class resistance gene relative abundance; “MC”, macrolide class resistance gene relative abundance; “LC”, lincosamide class resistance gene relative abundance; “NI”, nitroimidazole class resistance gene relative abundance; “PC”, phenicol class resistance gene relative abundance; “QL”, quinolone class resistance gene relative abundance; “TC”, tetracycline class resistance gene relative abundance.

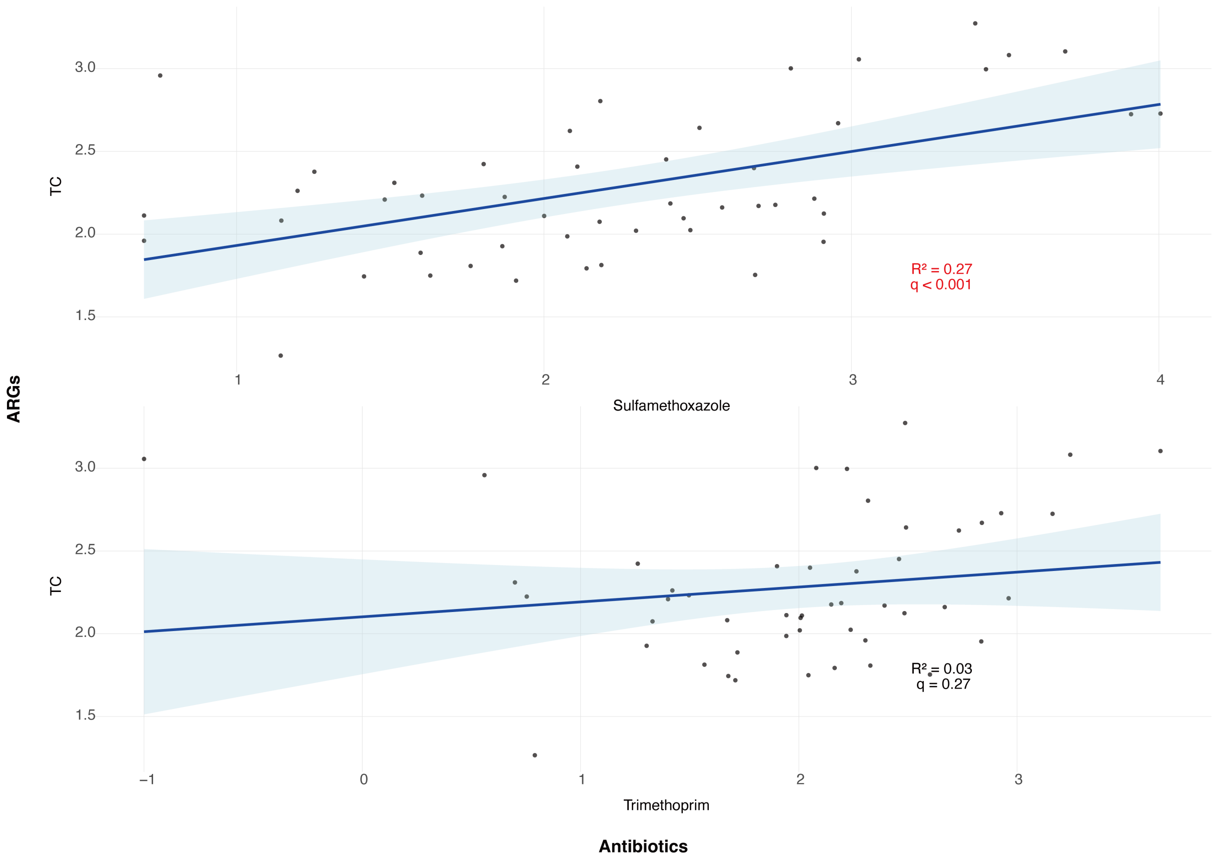

Supplementary Fig. 11 | Regression analysis of folate pathway antagonist concentrations against tetracycline class resistance gene relative abundance in globally sourced municipal wastewater samples. Statistical significance is determined using Benjamini-Hochberg controlled *p* values (denoted as *q* values) at a threshold of < 0.05. Significant associations ($q < 0.05$) are highlighted in red text in the scatterplots. Data presented here have been log10-transformed.

Abbreviation

"TC", tetracycline class resistance gene relative abundance.

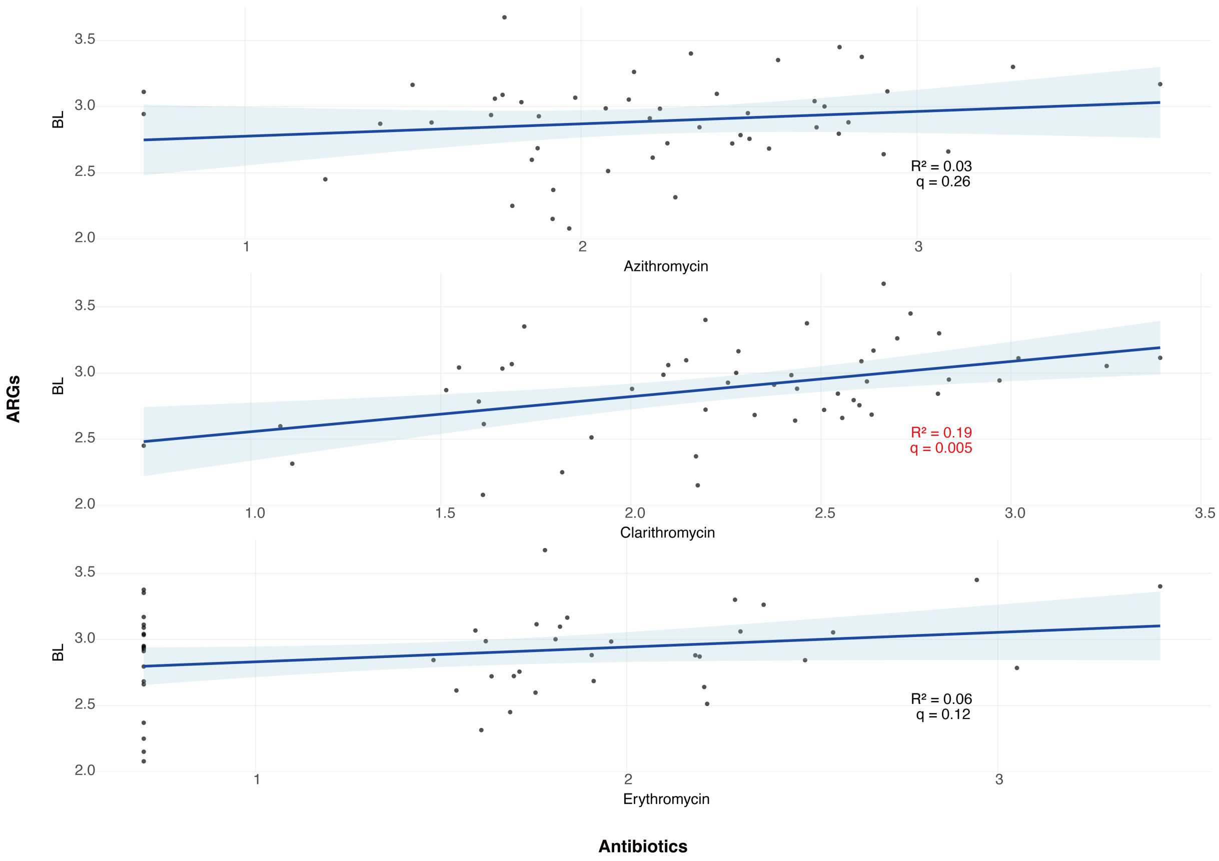

Supplementary Fig. 12 | Regression analysis of macrolide concentrations against beta-lactam class resistance gene relative abundance in globally sourced municipal wastewater samples.

Statistical significance is determined using Benjamini-Hochberg controlled *p* values (denoted as *q* values) at a threshold of < 0.05. Significant associations ($q < 0.05$) are highlighted in red text in the scatterplots. Data presented here have been log10-transformed.

Abbreviation

"BL", beta-lactam class resistance gene relative abundance.

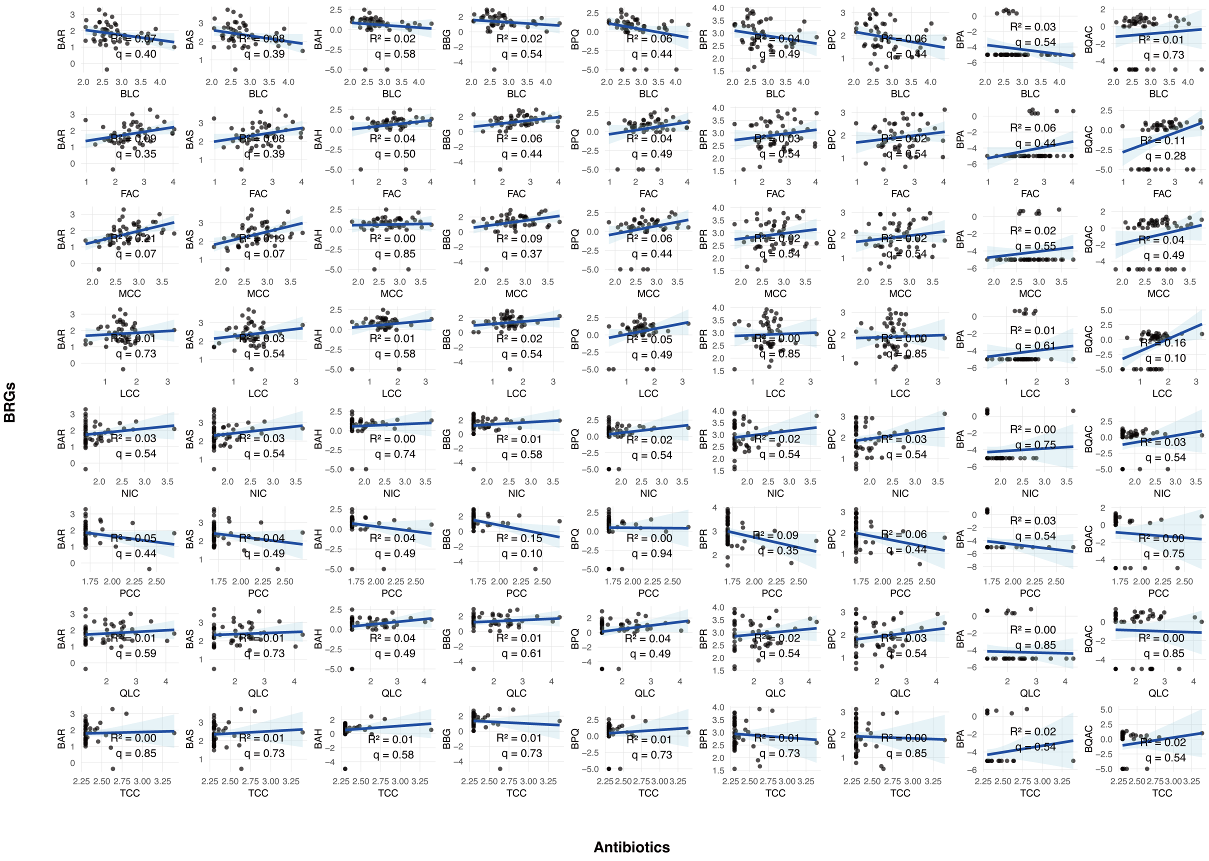

Supplementary Fig. 13 | Regression analysis of antibiotic concentrations (by class) against BRG relative abundances (by class) in globally sourced municipal wastewater samples.

Statistical significance is determined using Benjamini-Hochberg controlled *p* values (denoted as *q* values) at a threshold of < 0.05. Significant associations ($q < 0.05$) are highlighted in red text in the scatterplots. Data presented here have been log10-transformed.

Abbreviations

1. Antibiotic concentrations: "BLC", beta-lactam class antibiotic concentration; "FAC", folate pathway antagonist class antibiotic concentration; "MCC", macrolide class antibiotic concentration; "LCC", lincosamide class antibiotic concentration; "NIC", nitroimidazole class antibiotic concentration; "PCC", phenicol class antibiotic concentration; "QLC", quinolone class antibiotic concentration; "TCC", tetracycline class antibiotic concentration.

2. BRG relative abundances: “BAR”, biocide acetate class resistance gene relative abundance; “BAS”, biocide acid class resistance gene relative abundance; “BAH”, biocide aldehyde class resistance gene relative abundance; “BBG”, biocide biguanide class resistance gene relative abundance; “BPQ”, biocide paraquat class resistance gene relative abundance; “BPR”, biocide class resistance gene relative abundance; “BPC”, biocide phenolic compound class resistance gene relative abundance; “BPA”, biocide polyamine class resistance gene relative abundance; “BQAC”: biocide quaternary ammonium compound class resistance gene relative abundance.

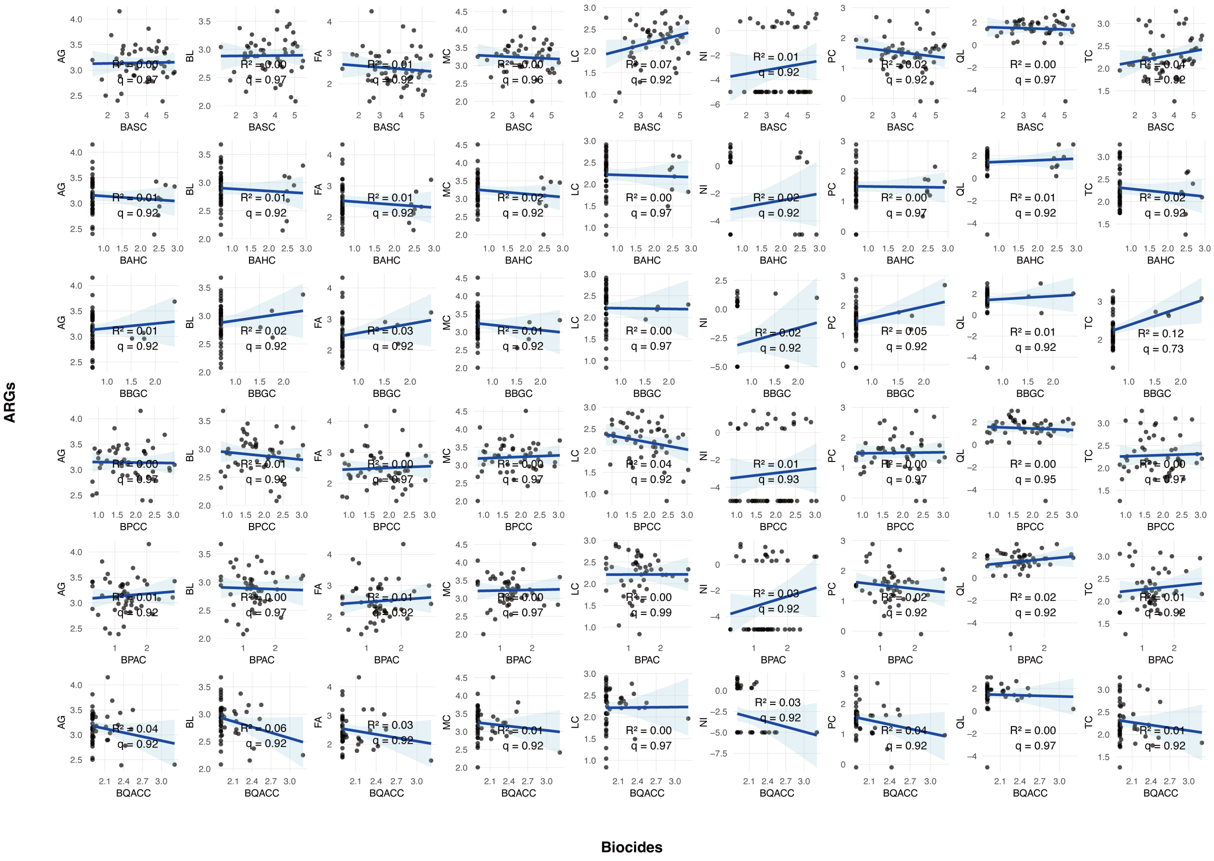

Supplementary Fig. 14 | Regression analysis of biocide concentrations (by class) against ARG relative abundances (by class) in globally sourced municipal wastewater samples.

Statistical significance is determined using Benjamini-Hochberg controlled *p* values (denoted as *q* values) at a threshold of < 0.05. Significant associations ($q < 0.05$) are highlighted in red text in the scatterplots. Data presented here have been log10-transformed.

Abbreviations

1. Biocide concentrations: “BASC”, biocide acid class concentration; “BAHC”, biocide aldehyde class concentration; “BBGC”, biocide biguanide class concentration; “BPCC”, biocide phenolic compound class concentration; “BPAC”, biocide polyamine class concentration; “BQACC”, biocide quaternary ammonium compound class concentration.

2. ARG relative abundances: “AG”: aminoglycoside class resistance gene relative abundance; “BL”, beta-lactam class resistance gene relative abundance; “FA”, folate pathway antagonist class resistance gene relative abundance; “MC”, macrolide class resistance gene relative abundance; “LC”, lincosamide class resistance gene relative abundance; “NI”, nitroimidazole class resistance gene relative abundance; “PC”, phenicol class resistance gene relative abundance; “QL”, quinolone class resistance gene relative abundance; “TC”, tetracycline class resistance gene relative abundance.

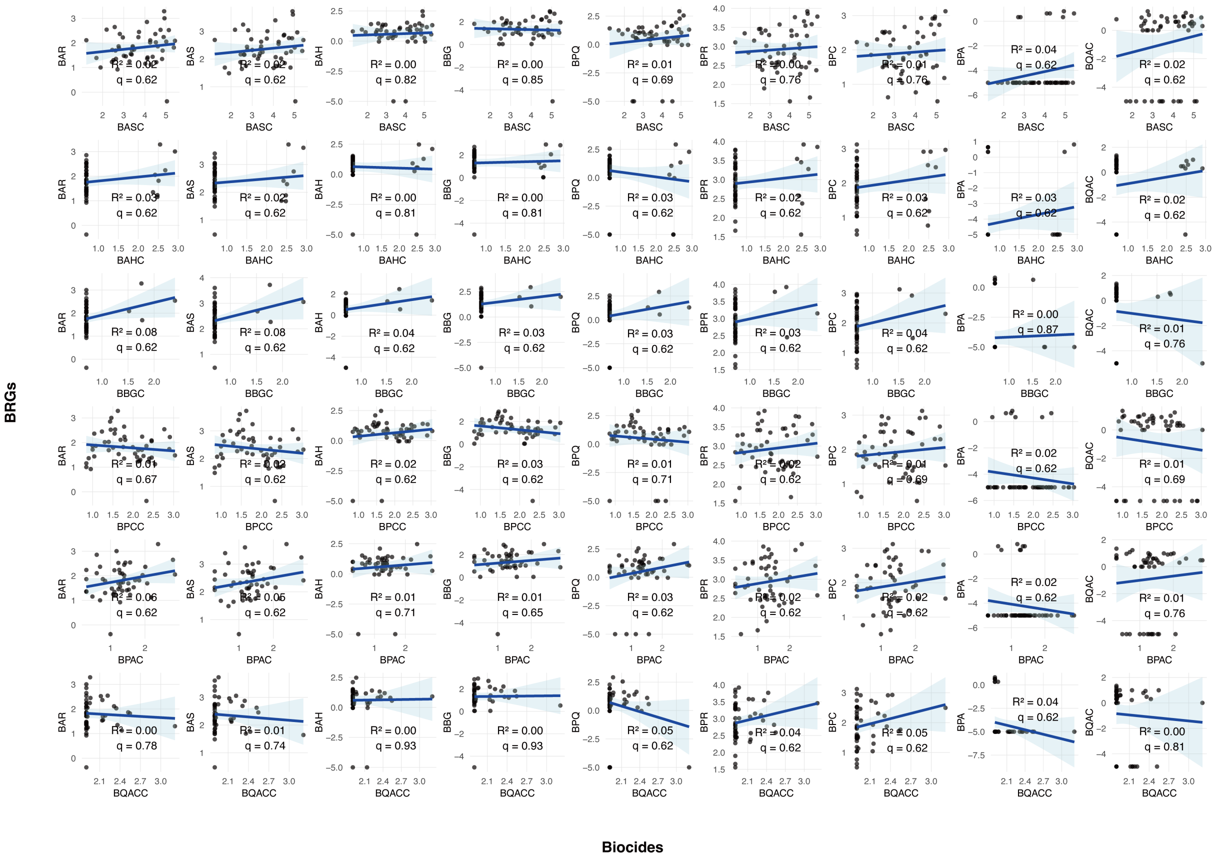

Supplementary Fig. 15 | Regression analysis of biocide concentrations (by class) against BRG relative abundances (by class) in globally sourced municipal wastewater samples.

Statistical significance is determined using Benjamini-Hochberg controlled *p* values (denoted as *q* values) at a threshold of < 0.05. Significant associations ($q < 0.05$) are highlighted in red text in the scatterplots. Data presented here have been log10-transformed.

Abbreviations

1. Biocide concentrations: “BASC”, biocide acid class concentration; “BAHC”, biocide aldehyde class concentration; “BBGC”, biocide biguanide class concentration; “BPCC”, biocide phenolic compound class concentration; “BPAC”, biocide polyamine class concentration; “BQACC”, biocide quaternary ammonium compound class concentration.

2. BRG relative abundances: “BAR”, biocide acetate class resistance gene relative abundance; “BAS”, biocide acid class resistance gene relative abundance; “BAH”, biocide aldehyde class resistance gene relative abundance; “BBG”, biocide biguanide class resistance gene relative abundance; “BPQ”, biocide paraquat class resistance gene relative abundance; “BPR”, biocide class resistance gene relative abundance; “BPC”, biocide phenolic compound class resistance gene relative abundance; “BPA”, biocide polyamine class resistance gene relative abundance; “BQAC”: biocide quaternary ammonium compound class resistance gene relative abundance.

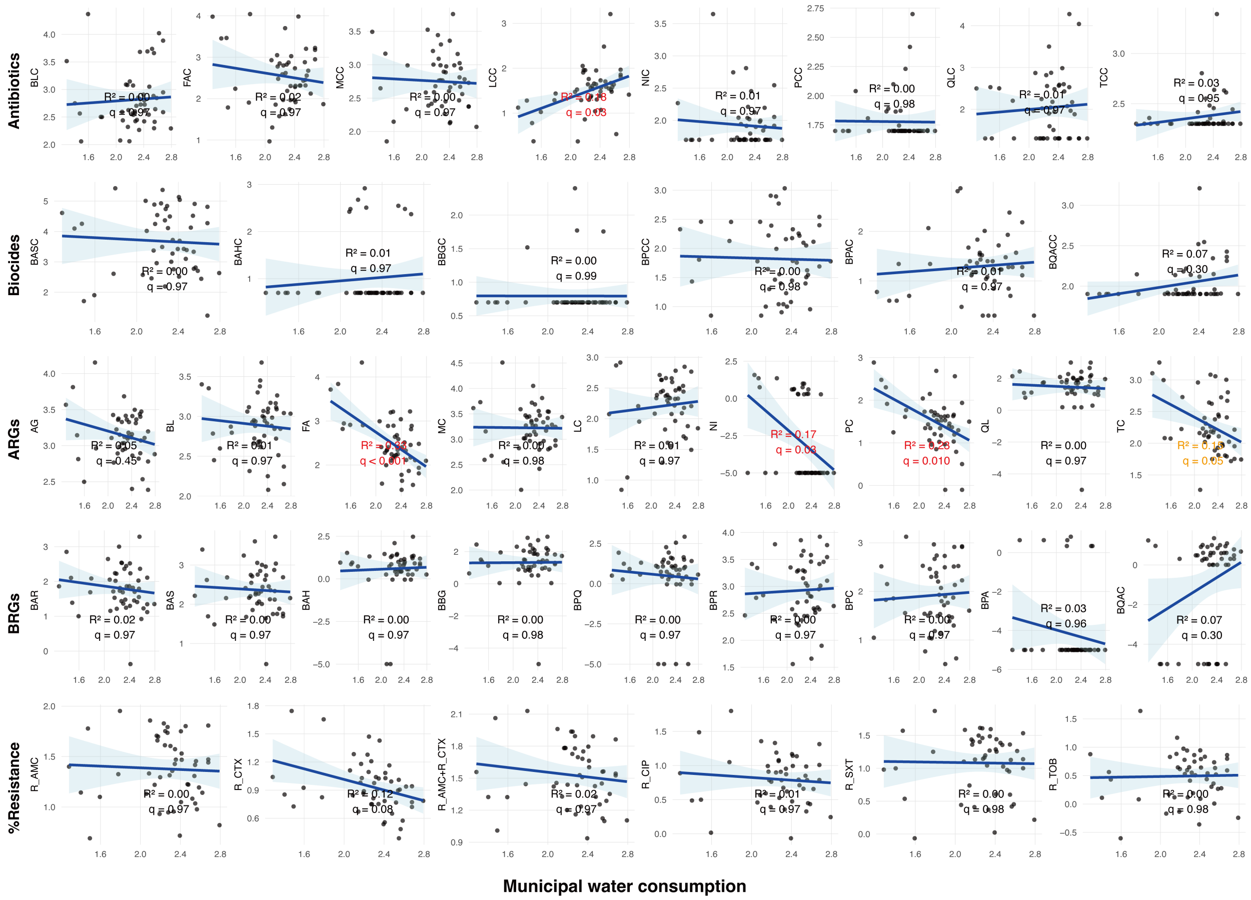

Supplementary Fig. 16 | Regression analysis of municipal water consumption levels against other variables. Statistical significance is determined using Benjamini-Hochberg controlled *p* values (denoted as *q* values) at a threshold of < 0.05. Significant associations ($q < 0.05$) are highlighted in red text in the scatterplots. Associations at the threshold ($q = 0.05$) are highlighted in orange text, representing findings at the borderline of statistical significance. Data presented here have been log10-transformed.

Abbreviations

1. Municipal water consumption: "wat_cons", municipal water consumption levels.

2. Antibiotic concentrations: "BLC", beta-lactam class antibiotic concentration; "FAC", folate pathway antagonist class antibiotic concentration; "MCC", macrolide class antibiotic concentration; "LCC", lincosamide class antibiotic concentration; "NIC", nitroimidazole class antibiotic concentration; "PCC", phenicol class

antibiotic concentration; “QLC”, quinolone class antibiotic concentration; “TCC”, tetracycline class antibiotic concentration.

3. Biocide concentrations: “BASC”, biocide acid class concentration; “BAHC”, biocide aldehyde class concentration; “BBGC”, biocide biguanide class concentration; “BPCC”, biocide phenolic compound class concentration; “BPAC”, biocide polyamine class concentration; “BQACC”, biocide quaternary ammonium compound class concentration.

4. ARG relative abundances: “AG”: aminoglycoside class resistance gene relative abundance; “BL”, beta-lactam class resistance gene relative abundance; “FA”, folate pathway antagonist class resistance gene relative abundance; “MC”, macrolide class resistance gene relative abundance; “LC”, lincosamide class resistance gene relative abundance; “NI”, nitroimidazole class resistance gene relative abundance; “PC”, phenicol class resistance gene relative abundance; “QL”, quinolone class resistance gene relative abundance; “TC”, tetracycline class resistance gene relative abundance.

5. BRG relative abundances: “BAR”, biocide acetate class resistance gene relative abundance; “BAS”, biocide acid class resistance gene relative abundance; “BAH”, biocide aldehyde class resistance gene relative abundance; “BBG”, biocide biguanide class resistance gene relative abundance; “BPQ”, biocide paraquat class resistance gene relative abundance; “BPR”, biocide class resistance gene relative abundance; “BPC”, biocide phenolic compound class resistance gene relative abundance; “BPA”, biocide polyamine class resistance gene relative abundance; “BQAC”: biocide quaternary ammonium compound class resistance gene relative abundance.

6. Selection potential (%resistance): “R_AMC”, observed %resistance after exposure for amoxicillin/clavulanic acid; “R_CTX”, observed %resistance after exposure for cefotaxime; “R_AMC+R_CTX”, sum of observed %resistance after exposure for amoxicillin/clavulanic acid and cefotaxime; “R_CIP”, observed %resistance after exposure for ciprofloxacin; “R_SXT”: observed %resistance after exposure for sulfamethoxazole/trimethoprim; “R_TOB”, observed %resistance after exposure for tobramycin.

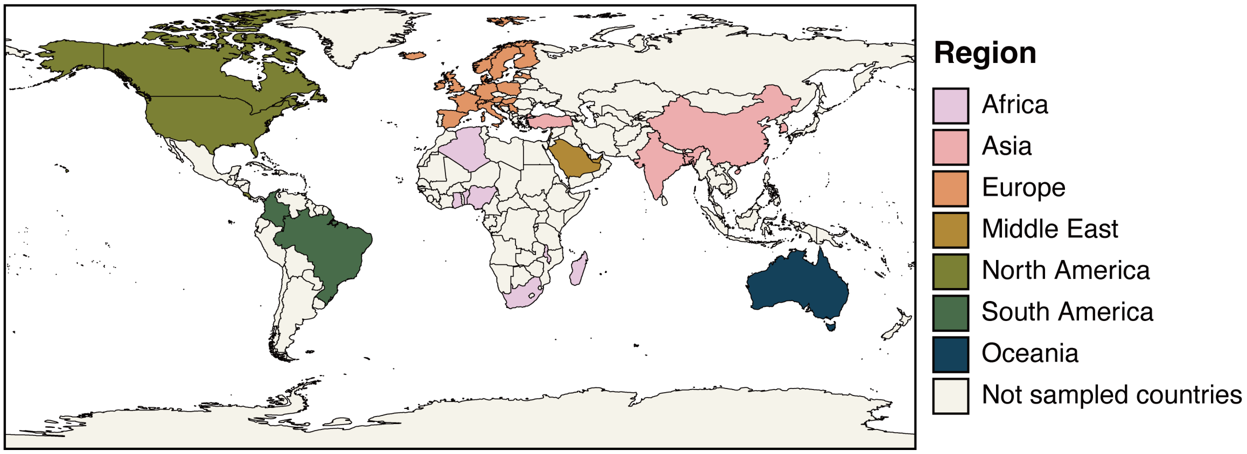

Supplementary Fig. 17 | Origins of the analyzed wastewater samples.

The world map was created in R (v. 4.4.3) using ggplot2 (v. 4.0.0) [2] with country boundary data obtained from Natural Earth (https://www.naturalearthdata.com/) via the rnaturalearth (v. 1.1.0) [3] rnaturalearthdata (v. 1.0.0) [4] packages. Country boundaries were retrieved at medium scale (1:50m resolution). Countries were color-coded by continent to visualize the geographic distribution of the studied wastewater samples. Three municipal wastewater samples were collected from locations in one country (the United States of America), namely Christiansburg (Virginia), Seattle (Washington), and South Milwaukee (Wisconsin). For all other countries in the study, a single sample was collected per country. All samples were collected in a campaign as part of the Global Sewage Surveillance Project [5] (see the main article for the updated reference of this citation), Supplementary Data 8.

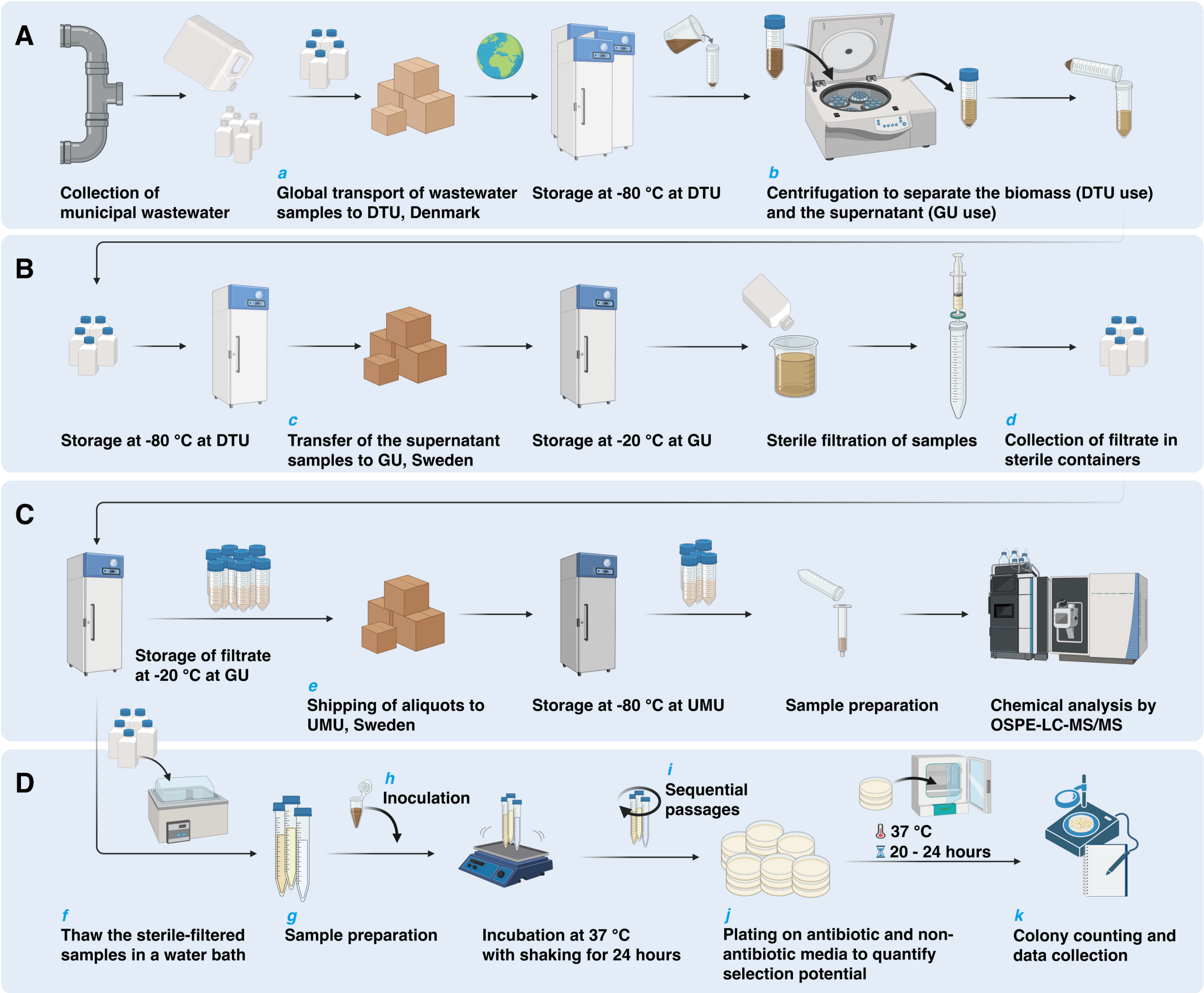

Supplementary Fig. 18 | Outline of sample processing and selection assay.

A Initial Sample Collection and Pre-processing. ***a*** Wastewater samples from 47 countries were collected, frozen, and transported to DTU for processing according to a standardized protocol [6]. The municipal wastewater samples (n = 49) were stored at -80 °C. ***b*** During sample processing, centrifugation at 10,000 *g* for 10 min was performed to separate the biomass (used at DTU: DNA extraction and metagenomic analysis, etc.) from the supernatant (reserved for GU: sterile-filtration and selection test, etc.). Both the biomass and the supernatant were stored at -80 °C at DTU before further experiments.

B Sterile Filtration. ***c*** Frozen supernatant samples were transferred to GU and stored at -20 °C. ***d*** Samples underwent sterile filtration through 0.22 μm filters. Filtrate aliquots were stored at -20 °C.

C Chemical Analysis. ***e*** A subset of the filtrate aliquots was shipped to UMU and stored at -80 °C. After sample

preparation, OSPE-LC-MS/MS was used to quantify the chemical concentrations.

D Selection Experiments. ***f*** Sterile-filtered wastewater samples were thawed in a water bath. ***g*** Samples were adjusted to have a final concentration of 10% LB. ***h*** Either a synthetic *E. coli* community or a natural wastewater community was added as an inoculant (final *E. coli* population density: ca 5×10^5 CFU/mL). Testing media were incubated at 37 °C with shaking for 24 hours. ***i*** Sequential passages were performed (72 hours total). ***j*** Selection potential was assessed by plating on antibiotic-containing and non-antibiotic media. ***k*** Colony-forming units (CFUs) data were collected for downstream analysis. In the plating, cation-adjusted Mueller-Hinton or CHROMagar™ ECC plates were used for the synthetic *E. coli* community and the natural wastewater community, respectively.

Timeline Sample collection: April to July 2021. Global wastewater samples received at DTU: April to September 2021. Frozen supernatant sample received at GU: June 2022. Sterile filtration: July 2022. Chemical analysis: April to May 2024. Selection experiments: December 2023 to May 2024.

Abbreviations DTU: National Food Institute, Technical University of Denmark. GU: Department of Infectious Diseases, University of Gothenburg, Sweden. UMU: Department of Chemistry, Umea University, Sweden.

Supplementary Fig. 18 was created in BioRender. Larsson, J. (2025) https://BioRender.com/bccwvjn

Supplementary Text 1 | Resistance gene quantification.

Raw sequencing reads from metagenomic sequencing analysis of the globally sourced municipal wastewater samples (the same samples used in the present study prior to sterile filtration; available in the European Nucleotide Archive, https://www.ebi.ac.uk/ena/browser/view/PRJEB84064) underwent the ARGprofiler [7] pipeline for quality trimming via fastp [8]. Trimmed reads were aligned to the PanRes [9] database collection using the KMA algorithm [10]. Specifically, antibiotic resistance genes (ARGs) and biocide resistance genes (BRGs) were identified using the ResFinder [11] and MEGARes [12] databases, respectively. Resistance gene quantification was normalized by dividing aligned read counts by the corresponding reference template length (in Kb). Relative abundances were established by normalizing these length-adjusted gene counts against total bacterial read abundance, which was determined through KMA [10] mapping of sample reads to mOTU [13] reference sequences. Final analysis aggregated individual gene counts into broader resistance classifications.

For more detailed metagenomic sequencing methodologies and sequencing data analysis, refer to the studies by Munk et al. [14] and Martiny et al. [5]. Processed metagenomic data have been deposited in Zenodo (https://doi.org/10.5281/zenodo.14652833) [15].

References

1. Stanton, I. C., Murray, A. K., Zhang, L., Snape, J. & Gaze, W. H. Evolution of antibiotic resistance at low antibiotic concentrations including selection below the minimal selective concentration. *Commun Biol* **3**, 467 (2020).
2. Wickham, H. *ggplot2: Elegant Graphics for Data Analysis*. (Springer, New York, 2016).
3. Massicotte, P. & South, A. rnaturalearth: World map data from Natural Earth. *R package version 1.1.0.9000* https://docs.ropensci.org/rnaturalearth/ (2025).
4. South, A., Michael, S. & Massicotte, P. rnaturalearthdata: World vector map data from Natural Earth used in 'rnaturalearth'. *R package version 1.0.0.9000* https://docs.ropensci.org/rnaturalearthdata/ (2025).
5. Martiny, H.-M. *et al.* Geographics and bacterial networks differently shape the acquired and latent global sewage resistomes. *Nat Commun* (2025).
6. Hendriksen, R. S. *et al.* Global monitoring of antimicrobial resistance based on metagenomics analyses of urban sewage. *Nat Commun* **10**, 1124 (2019).
7. Martiny, H. M. *et al.* ARGprofiler—a pipeline for large-scale analysis of antimicrobial resistance genes and their flanking regions in metagenomic datasets. *Bioinformatics* **40**, (2024).
8. Chen, S. Ultrafast one-pass FASTQ data preprocessing, quality control, and deduplication using fastp. *iMeta* **2**, (2023).
9. Martiny, H. M. *et al.* PanRes - Collection of antimicrobial resistance genes. https://zenodo.org/records/10091602 (2023).
10. Clausen, P. T. L. C., Aarestrup, F. M. & Lund, O. Rapid and precise alignment of raw reads against redundant databases with KMA. *BMC Bioinformatics* **19**, (2018).
11. Bortolaia, V. *et al.* ResFinder 4.0 for predictions of phenotypes from genotypes. *J Antimicrob Chemother* **75**, 3491–3500 (2020).
12. Bonin, N. *et al.* MEGARes and AMR++, v3.0: An updated comprehensive database of antimicrobial resistance determinants and an improved software pipeline for classification using high-throughput sequencing. *Nucleic Acids Res* **51**, D744–D752 (2023).
13. Ruscheweyh, H. J. *et al.* Cultivation-independent genomes greatly expand taxonomic-profiling capabilities of mOTUs across various environments. *Microbiome* **10**, (2022).
14. Munk, P. *et al.* Genomic analysis of sewage from 101 countries reveals global landscape of antimicrobial resistance. *Nat Commun* **13**, 7251 (2022).
15. Martiny, H.-M., Munk, P., Fuschi, A. & Aarestrup, F. Data for Global Sewage 3 [Data set]. *Zenodo* https://doi.org/10.5281/zenodo.14652833 (2025).
