# Supplementary material for: Antibiotic resistance selection and deselection in municipal wastewater from 47 countries
Source: Nat Commun. 2025 Nov 3;16:9698. doi: 10.1038/s41467-025-65670-7 (PMC12583516; doi:10.1038/s41467-025-65670-7)
Supplement: Supplementary file 2 — Description of Additional Supplementary Files [file 41467_2025_65670_MOESM2_ESM.pdf]

Description of Additional Supplementary Files

Description for the Supplementary Dataset

File Name: Supplementary Data 1
Description: Comprehensive statistics for the synthetic *E. coli* community assay (saline, 0-hour reference).

File Name: Supplementary Data 2
Description: Comprehensive statistics for the synthetic *E. coli* community assay (saline, 72-hour reference).

File Name: Supplementary Data 3
Description: Comprehensive statistics for the natural wastewater community assay (saline, 0-hour reference).

File Name: Supplementary Data 4
Description: Selection potential (%resistance) and antibiotic concentrations of the samples from the sample processing and storage effects assessment.

File Name: Supplementary Data 5
Description: MIC distributions for *Escherichia coli* (EUCAST, database version 2024-05-13).

File Name: Supplementary Data 6
Description: Antibiotic concentrations of the globally sourced municipal wastewater samples.

File Name: Supplementary Data 7
Description: Antibacterial biocide concentrations of the globally sourced municipal wastewater samples.

File Name: Supplementary Data 8
Description: Sample list and information on the globally sourced municipal wastewater samples.

File Name: Supplementary Data 9
Description: Clinical breakpoints concentrations (EUCAST, database version 14.0).

File Name: Supplementary Data 10
Description: General information for the OSPE-LC-MS/MS analysis.

File Name: Supplementary Data 11
Description: Analyte MS specifics.

File Name: Supplementary Data 12
Description: MS specifics for internal standards.

File Name: Supplementary Data 13
Description: CAS information for the studied chemicals.

File Name: Supplementary Data 14
Description: Additional OSPE properties for the antibacterial biocide analysis.

File Name: Supplementary Data 15
Description: Municipal water consumption data of countries contributing to the global wastewater sampling.

File Name: Supplementary Data 16
Description: Metadata for the regression analysis of relating chemical concentrations to selection potential and resistance genes.

File Name: Supplementary Data 17
Description: Source data (CFU/mL reads on plates) for the synthetic *E. coli* community assay.

File Name: Supplementary Data 18
Description: Source data (CFU/mL reads on plates) for the natural wastewater community assay.
