# Supplementary material for: Antibiotic resistance selection and deselection in municipal wastewater from 47 countries
Source: Nat Commun. 2025 Nov 3;16:9698. doi: 10.1038/s41467-025-65670-7 (PMC12583516; doi:10.1038/s41467-025-65670-7)
Supplement: Supplementary file 4 — Reporting Summary [file 41467_2025_65670_MOESM4_ESM.pdf]

Corresponding author(s): D.G.Joakim Larsson
Last updated by author(s): Oct 20, 2025

Reporting Summary

Nature Portfolio wishes to improve the reproducibility of the work that we publish. This form provides structure for consistency and transparency in reporting. For further information on Nature Portfolio policies, see our Editorial Policies and the Editorial Policy Checklist.

Statistics

For all statistical analyses, confirm that the following items are present in the figure legend, table legend, main text, or Methods section.

n/a	Confirmed	
☐	☒	The exact sample size (*n*) for each experimental group/condition, given as a discrete number and unit of measurement
☐	☒	A statement on whether measurements were taken from distinct samples or whether the same sample was measured repeatedly
☐	☒	The statistical test(s) used AND whether they are one- or two-sided<br>*Only common tests should be described solely by name; describe more complex techniques in the Methods section.*
☐	☒	A description of all covariates tested
☐	☒	A description of any assumptions or corrections, such as tests of normality and adjustment for multiple comparisons
☐	☒	A full description of the statistical parameters including central tendency (e.g. means) or other basic estimates (e.g. regression coefficient) AND variation (e.g. standard deviation) or associated estimates of uncertainty (e.g. confidence intervals)
☐	☒	For null hypothesis testing, the test statistic (e.g. *F*, *t*, *r*) with confidence intervals, effect sizes, degrees of freedom and *P* value noted<br>*Give P values as exact values whenever suitable.*
☒	☐	For Bayesian analysis, information on the choice of priors and Markov chain Monte Carlo settings
☒	☐	For hierarchical and complex designs, identification of the appropriate level for tests and full reporting of outcomes
☐	☒	Estimates of effect sizes (e.g. Cohen's *d*, Pearson's *r*), indicating how they were calculated

Our web collection on statistics for biologists contains articles on many of the points above.

Software and code

Policy information about availability of computer code

Data collection	No software was used to collect data.
Data analysis	The code for the core statistical analysis of selection potential is provided on the github page https://github.com/watNoel/Global-Sewage-Project. For this analysis, versions were:<br>R (4.4.0)<br>MASS (version 7.3-60.2)<br>stats (version 4.4.0)<br>multcomp (version 1.4-25)<br>emmeans (version 1.10.3)<br>tidyverse (version 2.0.0)<br>broom (version 1.0.6)<br>magrittr version (2.0.3)<br>readxl (version 1.4.3)<br>writexl(version 1.5.0)<br><br>R packages and versions used in the analysis of effects of sample processing, and associations between ARGs,BRGs and selection potential are:<br>R (4.4.3)<br>rstatix (version 0.7.2)

emmeans (version 1.8.0)

Python and Python packages used to obtain metagenomic data, e.g antibiotic and biocide resistance gene/class counts, from previously published data sources were:
Python (3.12)
Pandas (version 2.3.2)

Additional R packages and versions used in the visualization of the origins of the analyzed wastewater samples are
R (4.4.3)
ggplot2 (version 4.0.0)
rnaturalearth (version 1.1.0)
rnaturalearthdata (version 1.0.0)

For manuscripts utilizing custom algorithms or software that are central to the research but not yet described in published literature, software must be made available to editors and reviewers. We strongly encourage code deposition in a community repository (e.g. GitHub). See the Nature Portfolio guidelines for submitting code & software for further information.

Data

Policy information about availability of data

All manuscripts must include a data availability statement. This statement should provide the following information, where applicable:

- Accession codes, unique identifiers, or web links for publicly available datasets
- A description of any restrictions on data availability
- For clinical datasets or third party data, please ensure that the statement adheres to our policy

The data generated in this study are provided in the 'Supplementary Dataset' file.

Data, including the raw data (CFU/mL reads on plates) from the synthetic and natural community assays, statistical analysis of selection potential, antibiotic and antibacterial biocide concentrations, as well as the ARG and BRG resistance gene relative abundances (in log10), are provided in the Supplementary Dataset.

The metagenomic data pertaining to samples in this study are available at the European Nucleotide Archive with accession code PRJEB84064 (https://www.ebi.ac.uk/ena/browser/view/PRJEB84064). Processed metagenomic data used in the study are available on Zenodo (https://doi.org/10.5281/zenodo.14652833).

Research involving human participants, their data, or biological material

Policy information about studies with human participants or human data. See also policy information about sex, gender (identity/presentation), and sexual orientation and race, ethnicity and racism.

Reporting on sex and gender	Not applicable.
Reporting on race, ethnicity, or other socially relevant groupings	Not applicable.
Population characteristics	Not applicable.
Recruitment	Not applicable.
Ethics oversight	Not applicable.

Note that full information on the approval of the study protocol must also be provided in the manuscript.

Field-specific reporting

Please select the one below that is the best fit for your research. If you are not sure, read the appropriate sections before making your selection.

[x] Life sciences [ ] Behavioural & social sciences [ ] Ecological, evolutionary & environmental sciences

For a reference copy of the document with all sections, see nature.com/documents/nr-reporting-summary-flat.pdf

Life sciences study design

All studies must disclose on these points even when the disclosure is negative.

Sample size	The wastewater samples/countries included were selected were based on availability of sufficent volumes of left-over supernatants from a previous study coordinated by Frank Aarestrup.
Data exclusions	No countries/samples were exluded (see above)
Replication	There are 49 different municipal wastewaters included, but within each country there is just one sample (except for three from the USA). Hence, we refrain from drawing conclusion on differences between specific countries. Technical replicates were included, with selection

	experiments done on different days.
Randomization	There was no special order in which samples were tested, more than that one technical replicate was first analysed for all countries, then a second, then a third etc (on different days)
Blinding	The samples were not blinded (contry codes were available) but note that we are not testing any hypothesis of differences between countries. Readouts, whether assessment of peak sizes for chemical analyses or colony counts for selection experiments, were quantitative and with none or very limited subjective influence in comparison with the effect sizes.

Reporting for specific materials, systems and methods

We require information from authors about some types of materials, experimental systems and methods used in many studies. Here, indicate whether each material, system or method listed is relevant to your study. If you are not sure if a list item applies to your research, read the appropriate section before selecting a response.

Materials & experimental systems

n/a	Involved in the study
☒	☐ Antibodies
☒	☐ Eukaryotic cell lines
☒	☐ Palaeontology and archaeology
☒	☐ Animals and other organisms
☒	☐ Clinical data
☒	☐ Dual use research of concern
☒	☐ Plants

Methods

n/a	Involved in the study
☒	☐ ChIP-seq
☒	☐ Flow cytometry
☒	☐ MRI-based neuroimaging

Plants

Seed stocks	Not applicable.
Novel plant genotypes	Not applicable.
Authentication	Not applicable.
